# High-EPA Biomass from *Nannochloropsis salina* Cultivated in a Flat-Panel Photo-Bioreactor on a Process Water-Enriched Growth Medium

**DOI:** 10.3390/md14080144

**Published:** 2016-07-29

**Authors:** Hamed Safafar, Michael Z. Hass, Per Møller, Susan L. Holdt, Charlotte Jacobsen

**Affiliations:** 1 Technical University of Denmark, National Food Institute (DTU Food), Søltofts Plads, Building 221, Kongens Lyngby 2800, Denmark; hasaf@food.dtu.dk (H.S.); suho@food.dtu.dk (S.L.H.); 2 Kalundborg Municipality, Department Development, Torvet 3A, Kalundborg 4400, Denmark; michaelzhass@gmail.com (M.Z.H.); Per.Moller@kalundborg.dk (P.M.)

**Keywords:** EPA, *Nannochloropsis salina*, industrial process water, microalgae, carotenoids, amino acid, tocopherol, large scale, flat panel photo-bioreactor

## Abstract

*Nannochloropsis salina* was grown on a mixture of standard growth media and pre-gasified industrial process water representing effluent from a local biogas plant. The study aimed to investigate the effects of enriched growth media and cultivation time on nutritional composition of *Nannochloropsis salina* biomass, with a focus on eicosapentaenoic acid (EPA). Variations in fatty acid composition, lipids, protein, amino acids, tocopherols and pigments were studied and results compared to algae cultivated on F/2 media as reference. Mixed growth media and process water enhanced the nutritional quality of *Nannochloropsis salina* in laboratory scale when compared to algae cultivated in standard F/2 medium. Data from laboratory scale translated to the large scale using a 4000 L flat panel photo-bioreactor system. The algae growth rate in winter conditions in Denmark was slow, but results revealed that large-scale cultivation of *Nannochloropsis salina* at these conditions could improve the nutritional properties such as EPA, tocopherol, protein and carotenoids compared to laboratory-scale cultivated microalgae. EPA reached 44.2% ± 2.30% of total fatty acids, and α-tocopherol reached 431 ± 28 µg/g of biomass dry weight after 21 days of cultivation. Variations in chemical compositions of *Nannochloropsis salina* were studied during the course of cultivation. *Nannochloropsis salina* can be presented as a good candidate for winter time cultivation in Denmark. The resulting biomass is a rich source of EPA and also a good source of protein (amino acids), tocopherols and carotenoids for potential use in aquaculture feed industry.

## 1. Introduction

Microalgae are autotrophic microorganisms, which are able to produce biomass from solar energy, CO_2_ and nutrients, with higher photosynthetic activity compared to terrestrial plants [1,2]. Resulting biomass includes important metabolites such as carbohydrates, lipids, proteins and also many other bioactive compounds like pigments and phenolics. Microalgal biomass may be used for different applications, such as biofuel production, wastewater treatment, production and extraction of bioactive compounds, or as a food/feed ingredient [3,4]. Actually, one of the most promising applications of microalgae biomass is as feed for aquatic animals. Algae are utilized for various applications in aquaculture. The importance of microalgae in aquaculture food chains is mainly due to their fatty acid, carotenoids and protein (amino acid) composition [5,6]. Use of microalgae as a rich source of fatty acids for the aquaculture has become the focus of industrial and scientific developments. Microalgae suitable for utilization in aquaculture should possess certain important characteristics such as appropriate nutritional value, high production rates, suitability for mass cultivation, resistance to fluctuating growth conditions, and nontoxicity [7]. Species such as *Chlorella, Isochrysis*, *Pavlova*, *Phaeodactylum*, *Chaetoceros*, *Nannochloropsis*, and *Thalassiosira* are reported as the most frequent used [8].

*Nannochloropsis* is a genus comprised of small (less than 5 µm) coccoid uni cells and is known primarily from the marine environment [9]. Different species from the genus *Nannochloropsis* have received sustained interest due to their high biomass and lipid productivity and their potential for cultivation at a large scale [9]. Members of the genus *Nannochloropsis* (Eustigmatophyceae) are widely distributed in marine and fresh water ecosystems; and individually in coastal waters [9]. There are six recognized species in the *Nannochloropsis* genus as *Nannochloropsis gaditana*, *Nannochloropsis salina*, *Nannochloropsis granulata*, *Nannochloropsis limnetica*, *Nannochloropsis oceanica*, and *Nannochloropsis oculata* [9,10].

The microalga *Nannochloropsis salina* is one of the most promising candidates for large scale cultivation. On the other hand this microalga accumulate high amounts of eicosapentaenoic acid (EPA), which is one of the most favored fatty acids in aquaculture [11]. Several scientific investigations have been published regarding the biotechnological potential of *Nannochloropsis salina* [12,13,14,15,16]. Feasibility of large scale cultivation of *Nannochloropsis* sp. in photo bioreactors has been studied in several studies [16,17,18,19]. Zittelli et al. [17] cultivated *Nannochloropsis* sp. in a flat panel photo-bioreactor under artificial light and demonstrated that higher illumination increased the biomass productivity but decreased the content of pigments (Chlorophyll a and carotenoids). Variations in lipid classes and fatty acid composition were studied during 2007–2009 in *Nannochloropsis oculata* cultivated in vertical flat panel photo-bioreactors and explained by seasonal light and temperature [18].

The effect of light-path length in flat plate reactors on eicosapentaenoic acid, lipid and biomass productivity of outdoor cultivated *Nannochloropsis* sp. was tested by Zou et al. [19]. In this study, the optimal light-path for culturing *Nannochloropsis* was reported as ca. 10 cm. Olofsen et al. [20] investigated lipid and biomass productivity and fatty acid composition of *Nannochloropsis oculata* cultivated in flat panel photo-bioreactors under different nitrogen limitation conditions during autumn and spring. In this study, *Nannochloropsis occulata* demonstrated high biomass productivity under nitrogen limitation comparing to nitrogen starvation during both seasons. Eicosapentaenoic acid (EPA) production by *Nannochloropsis gaditana* in different pilot scale outdoor photo-bioreactors was investigated by Camacho-Rodríguez et al. [21]. In this study, the biomass productivity in a vertical flat-panel bioreactor (FP-PBR) was reported as 10-fold higher compared to the traditional production system. San Pedro et al. [22] studied the feasibility of growing *Nannochloropsis gaditana* in outdoors raceway ponds. In this study, growth and lipid production models in relation to operational and environmental conditions were also developed.

Large scale production of microalgae biomass faces several challenges to be economically feasible, and the cultivation cost is one of the major factors affecting the price of final product. One of the strategies to resolve this problem is to use a low-cost culture medium such as waste water [23].

Some species of microalgae are capable of growing in municipal, industrial and agricultural wastewater, producing biomass while reducing the load of nitrogen and phosphorus [24,25,26]. On the other hand, the wastewater composition should be free of pollutants such as heavy metals, when the resulting microalgal biomass is intended to be used as a feed ingredient. Trace metals at levels that do not inhibit algal growth still cause problems via their presence in algae biomass are used in aquaculture or for animal feedstocks [25]. Under anaerobic conditions, methanogenic conversion of organic matter to biogas will result in methane, CO_2_, and an effluent which contains ammonia. The conversion process, known as anaerobic digestion, is being done in an anaerobic sludge tower reactor with internal circulation (ICT), so the effluent is called IC water (ICW). The performance of a microalgae cultivated on ICW effluent depends on a variety of factors including characteristics of algal species, properties of the anaerobic digestion effluent, and operational parameters during algal growth [25,26].

The objective of the present study was to find an economically viable use of the industrial process water nutrients and to investigate the effects of ICW enriched growth media and cultivation time on nutritional composition of *Nannochloropsis salina* biomass, with a focus on eicosapentaenoic acid (EPA). The aim was to obtain an algal biomass, which potentially could be used as an ingredient for aquaculture feed. The reference standard culture medium used was the F/2 medium [27], which is widely used as enriched seawater medium for growing marine microalgae. The F/2 medium has a low concentration of ${\text{NO}}_{3}^{-}$ as nitrogen source (0.75 mM), so addition of another N source (ammonium) affects growth rate and chemical composition of resulting biomass. The optimal growth condition was determined via batch laboratory experiments in order to provide basic reference data to be used in large scale. The optimal growth conditions were subsequently validated in large scale. This study revealed changes in nutritional composition of biomass during the course of cultivation and demonstrated which harvest time would be the optimal.

## 2. Results and Discussion

### 2.1. Growth Rate in Laboratory Scale Experiments

**Figure 1:**
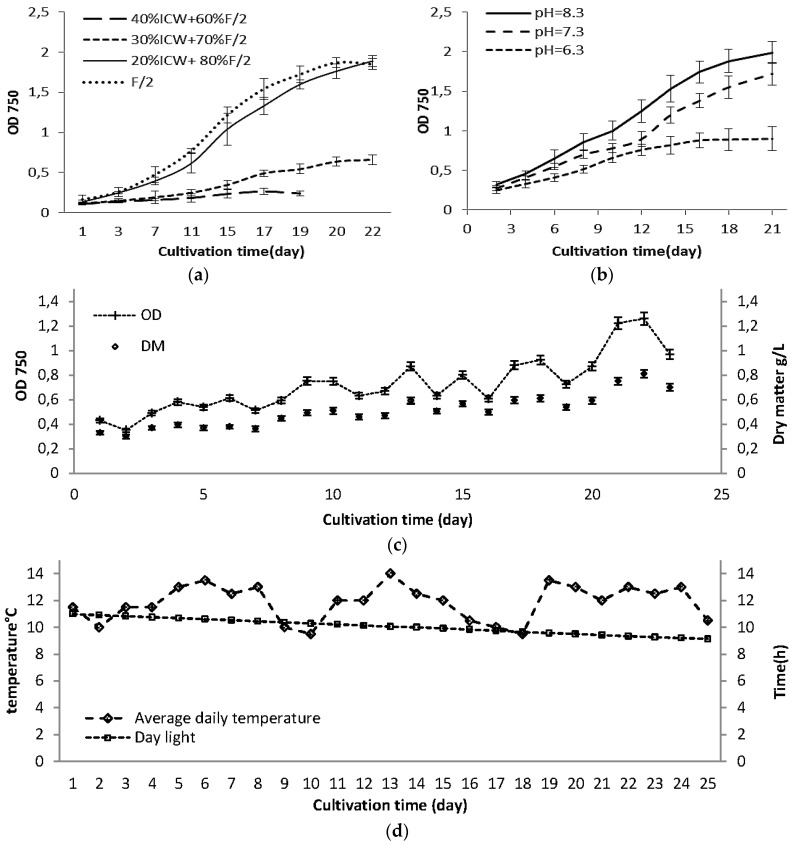
Growth curves for: (**a**) Effect of different levels of industrial process water on growth of *Nannochloropsis salina* at pH = 8.5; (**b**) *Nannochloropsis salina* cultivated on 20% ICW + 80% F/2 at 3 different pH; (**c**) large scale cultivated *Nannochloropsis salina*; and (**d**) variation in daily light hours and temperature during large scale cultivation. Large scale cultivation was done at pH = 8.3 ± 0.2 using 20% ICW + 80% F/2 as growth media at October–November 2015 in Kalundborg, Denmark. The standard errors are presented as bars (*n* = 2).

A number of nitrogen compounds are available for algae to assimilate: nitrate, nitrite, ammonium, and even organic nitrogen, and in general ammonium is the most preferable chemical form because it takes less energy for algae to assimilate into amino acids [28]. On the other hand, ammonium tolerance is different for various algae species and can be strongly influenced by the environmental conditions. Since free ammonia is toxic to algae, it can be stored in the system through an ammonia binding reaction [29]. In Figure 1a the effect of different levels of ICW on the growth rates of *Nannochloropsis salina* are shown. These observations showed that substitution of more than 20% of standard F/2 could retard the growth rate of *Nannochloropsis salina* at pH 8 and room temperature. Our preliminary observations (data not shown) revealed that *Nannochloropsis salina* cannot grow successfully on ICW as the main nutrient source. The nitrogen source in F/2 media is nitrate, but in process water mostly includes ammonia + ammonium-N (Table 1). The nitrogen-to-phosphorus (N/P) ratio was 15 for F/2 and 17.2 for process water, which were close to the ideal atomic ratio of 16 for *N. salina* [30]. Some previous studies reported that *Nannochloropsis* sp. can utilize ammonium as well as nitrate [14,24,25,28,29,30,31]. The growth interruption of *Nannochloropsis salina* on ICW as main nutrient source might be related to different factors including concentration of ammonia in growth media (Table 2). Ammonia toxicity is mainly attributed to NH_3_ at pH > 9 and at pH < 8, toxicity is likely associated with the ammonium ion rather than ammonia. During growth of algae on a growth media containing ammonium, the pH decreases, as H^+^ ions released to the medium. In contrast, growth on nitrate causes an increase in the pH due to the release of OH^−^ ions [31]. This phenomenon can result in less available carbon, as the main source of the carbon was CO_2_, which was injected automatically when the pH raised over set value (e.g., pH = 8). Cultivation of *Nannochloropsis salina* at various pH values (8.3 ± 0.2, 7.3 ± 0.2 and 6.3 ± 0.2) with the mixed nutrient composition (20% ICW + 80% F/2) showed that at pH 6.3, the growth rate was significantly lower than at higher pH (Figure 1b). It has also been reported previously that optimum pH for *Nannochloropsis salina* ranged from 7.5 to 8.5 [32]. In this study pH 8.3 ± 0.2 was selected as the optimum pH for further experiments.

### 2.2. Changes in Biomass Composition in Response to Growth Media

#### 2.2.1. Protein, Lipids and Tocopherols

**Figure 2:**
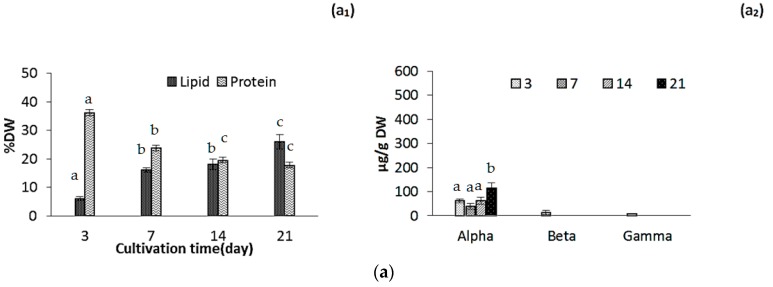

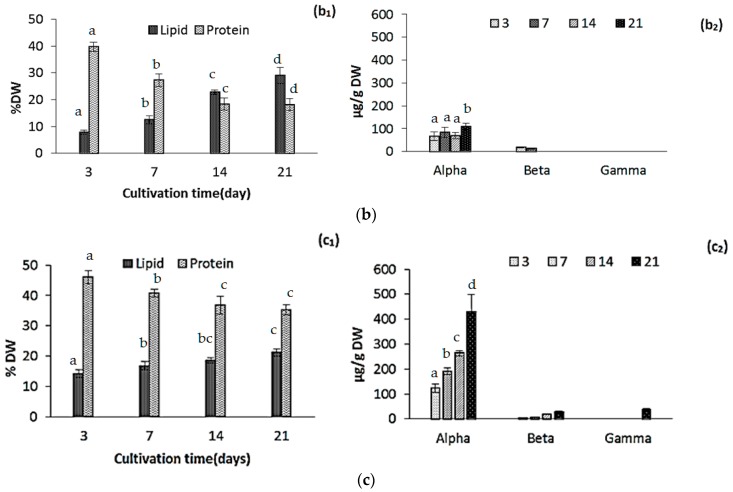
Lipid and protein content (**a_1_**,**b_1_**,**c_1_**) and tocopherol composition (**a_2_**,**b_2_**,**c_2_**) for (**a**) F/2 experiment; (**b**) ICW + F/2 experiment and (**c**) large scale experiment, respectively. The standard errors are presented as bars (*n* = 2), and different letters indicate significant differences (*p* < 0.05).

Lipid content increased during the cultivation from 7.9% ± 0.50% DW to 29.0% ± 1.50% DW and from 6.15% ± 1% DW to 26.0% ± 1.75% DW, for ICW + F/2 and F/2 cultivated microalgae, respectively (Figure 2a,b). Effect of growth media was evaluated as not significant while cultivation time significantly affected the oil accumulation in both experiments (*p* < 0.05). Amounts of lipids in biomass depend on several factors such as nutrient composition and environmental factors, so comparison of the results with other studies is not possible. Various amounts of lipids in *N. salina* were previously reported [11,12,14,24,33]. One study reported that under nitrate limitation, total fatty acids could exceed 70% of the biological dry mass in this specie [11], while in another study lipid accumulation in *N. salina* was the lowest (36.95% ± 0.91% DW) among 9 different *Nannochloropsis* species [12]. Several studies demonstrated that accumulation of lipids is enhanced in nitrogen-starved or deprived cultures of microalgae [11,14,32]. In our study, relatively high N/P ratio of the growth media may have impacted the lipid contents. However, our findings indicated that nitrogen starvation most likely had not occurred during cultivation for 21 days.

In general, microalgae have a limited ability to produce nitrogen storage materials when growing under nitrogen-sufficient conditions, with some exceptions in cyanobacteria. When microalgae are grown under nitrogen starvation, the most striking effect is the active and specific degradation of phycobilisomes [32]. Until available nitrogen in cell falls below a threshold value, photosynthesis still continues at lower rate. Under these circumstances, photosynthetically fixed carbon is then diverted from the protein synthesis into the pathways for carbohydrate and lipid synthesis [32], resulting in gradual decrease of protein during the cultivation. Our results confirm this phenomenon as protein content decreased during the cultivation from 39.7% DW to 18.1% DW and from 36.2% DW to 17.8% DW for ICW + F/2 and F/2 cultivated microalgae, respectively (Figure 2a_1_,b_1_). Both growth media and cultivation time had a statistically significant effect on protein content at the 95.0% confidence level (*p* < 0.05). Difference in protein contents was, however, not significant after day 14.

Tocopherol composition mostly included α-tocopherol as reported by several studies [34,35]. Total tocopherol content was not significantly different in ICW + F/2 and F/2 experiments during the cultivation (Figure 2a_2_,b_2_). Amounts of α-tocopherol ranged from 61.2 ± 8.4 µg/g to 113 ± 24 µg/g and 67.3 ± 8.5 µg/g to 109.2 ± 14 µg/g for ICW + F/2 and F/2 cultivated microalgae, respectively. The effects of cultivation time and growth media were not found statistically significant (*p* = 0.1034). Durmaz et al. [35] showed that decreasing nitrogen concentrations could lead to an increase in α-tocopherol accumulation in *N. oculata*. Concentration of α-tocopherol could be attributed to different factors such as; nitrogen source (e.g., ammonium or nitrate), nitrogen concentration, growth phase and light (L)/dark (D) photoperiod. Microalgae *Nannochloropsis* sp. produce significantly lower quantities of α-tocopherol at 12:12 h (L:D) photo period compared to 24:0 h (L:D) [35]. It could be a reason for lower amounts of α-tocopherol in our experiments compared to the values reported by Durmaz et al. [35].

#### 2.2.2. Fatty Acid Composition

The major fatty acids in *Nannochloropsis* sp. are 14:0, 16:0, 16:1, *n*-7 and EPA, but C18 fatty acids and 20:4 (*n*-6) are present in lower quantities [11,12,13,15]. Table 3 shows the variation of the fatty acid compositions of *N. salina* grown on two growth media. During the 21 days of cultivation, EPA increased significantly from 4.95% ± 0.78% TFA to 32.0% ± 0.82% TFA and from 7.47% ± 0.23% TFA to 37.1% ± 0.77% TFA, for ICW + F/2 and F/2 cultivated microalgae, respectively. Both cultivation time and growth media had a statistically significant effect on EPA variations at the 95.0% confidence level. Amounts of C 20:4 (*n*-6) were higher in F/2 cultivated microalgae during the course of cultivation. Palmitic acid was the major saturated fatty acid in both experiments. Highest amounts of palmitic acid were detected in samples from the 3rd day of cultivation, which decreased from 48.8% ± 2.59% TFA to 21.3% ± 0.30% TFA and from 43.3% ± 0.98% TFA to 19.1% ± 0.40% TFA after 21 days, for ICW + F/2 and F/2 cultivated microalgae, respectively. Only cultivation time had a statistically significant effect on variation of C16:0 at the 95.0% confidence level. C16:1 (*n*-7) was detected as the main *n*-7 fatty acid in *N. salina* in this study, and it decreased during the cultivation for F/2 cultivated microalgae, but variations were not significant. Effects of cultivation time and growth media on the variations of C18:1 (*n*-7) were not significant at 95.0% confidence level. Total saturates varied significantly from 51.6% ± 3.25% TFA to 25.2% ± 1.63% TFA and from 48.3% ± 2.33% TFA to 25.9% ± 0.62% TFA for ICW + F/2 and F/2 cultivated microalgae, respectively. Variation in total saturated fatty acids was attributed to the cultivation time while both cultivation time and growth media had a statistically significant effect on total *n*-3 PUFA content at the 95.0% confidence level. EPA belongs to a group of fatty acids that are part of the phospholipids, which serve as structural components in the cell wall. Under nutritional limitations, such as nitrogen, cells are unable to resynthesize them or keep the concentration of these components constant [15]. However, with adequate nutrition, cells are capable of synthesizing high amounts of energy rich PUFAs, such as EPA [36], so enrichment of growth media with nitrogen (ammonia) successfully enhanced the EPA contents in *N. salina*.

Hu et al. [13] showed an increase of the EPA concentration of *N. salina* in batch cultures supplied with both high and low nitrate concentrations, so effect of environmental factors such as light intensity and temperature should also be considered in development of EPA in this species. It is generally assumed that microalgae have common biosynthetic pathway for production of EPA, that is, desaturation of 18:2 (*n*-6) is conducted by either Δ6 or Δ15 (*n*-3) desaturase trails, resulting in either 18:3 (*n*-6) or 18:3 (*n*-3), which lead to 20:4 (*n*-6) or EPA, respectively [36]. In high-CO_2_-grown cells, 18:2 (*n*-6) was of higher relative amount, and available for Δ6 desaturation, which gave rise to an increased production of 18:3 (*n*-6) and subsequently 20:5 (*n*-3) [37]. In the present study, 18:3 (*n*-6) was not detected, suggesting rapid turnover of 18:3 (*n*-6) and higher contents of EPA in the presence of CO_2_ as described in previous studies [13,37].

#### 2.2.3. Amino Acid Composition

**Figure 3:**
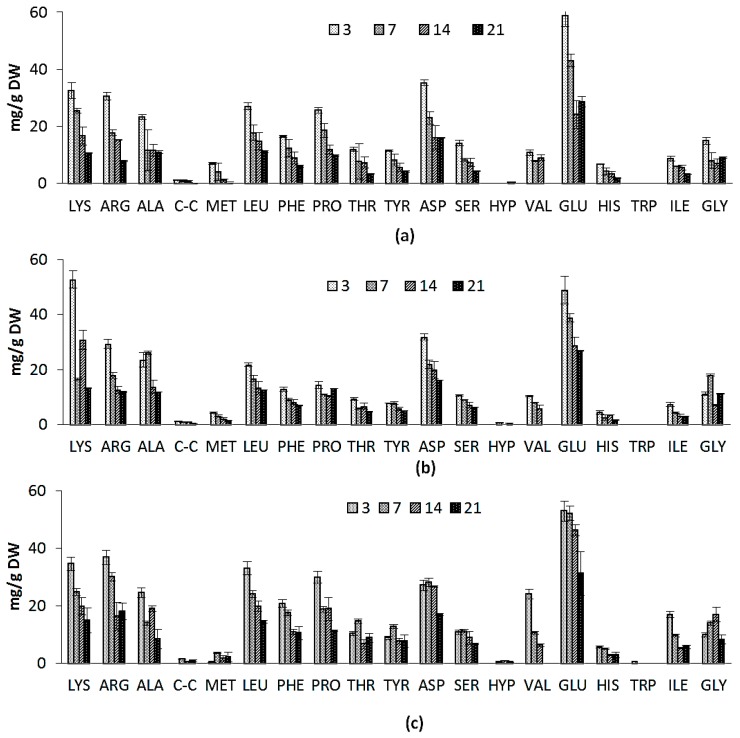

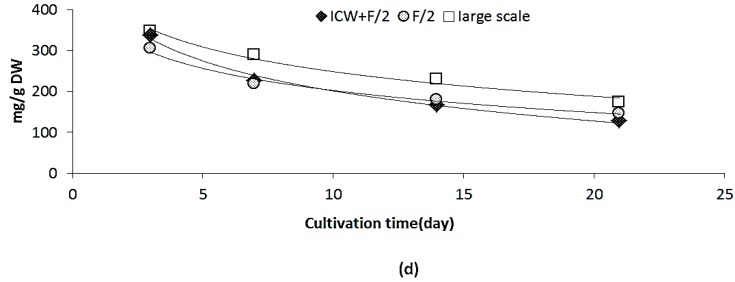
Amino acid composition for: (**a**) ICW + F/2 experiment; (**b**) F/2 experiment; (**c**) large scale experiment and (**d**) total amino acids variations during 21 days of cultivation for ICW + F/2, F/2 and large scale experiments. The standard errors are presented as bars (*n* = 2).

The amino-acid compositions of *Nannochloropsis salina* grown on two growth media is shown in Figure 3a,b. Nearly all of the amino acids were present in higher concentrations in ICW + F/2 cultivated samples. Concentration of amino acids decreased in the course of cultivation for both experiments. Aspartate, glutamate and arginine were generally found in the highest concentrations. Amino acid profiles are also similar to previous reports on *Nannochloropsis* genus and even microalgae species from different classes [38]. The similarity in the amino acid compositions suggests that the structure of microalgal protein may also be similar. This could indicate that many of the proteins performing specific functions and common to all species (e.g., C-fixation enzymes, membrane proteins associated with light-harvesting pigments) are highly conserved in their amino acid composition throughout the different species [38]. Notable difference was significantly higher amounts of lysine in F/2 cultivated microalgae samples at early stages of cultivation, while amounts of proline and glutamine were higher in ICW + F/2 cultivated samples. Ammonia is generally thought to directly assimilate into the amino acid glutamine [32], which can explain higher concentrations of glutamine in ICW + F/2 cultivated samples (58 mg/g vs. 48 mg/g). In general total amino acid content was higher in ICW + F/2 cultivated samples (Figure 3d), which could be contributed to higher amounts of nitrogen in growth media.

There was a statistically significant correlation at 95.0% confidence level (*p* < 0.05), between results of protein content and total amino acids in all experiments. The correlation coefficient equals 0.86, indicating a moderately strong relationship between the variables.

#### 2.2.4. Pigments

Pigment composition in *Nannochloropsis* sp. include chlorophyll a (not b or c), and also violaxanthin and vaucheriaxanthin esters, which play a major role in light harvesting as main accessory pigments [34,39]. Other carotenoids include xanthophylls such as canthaxanthin, anteraxanthin, zeaxanthin and carotenes such as β-carotene were also reported in *Nannochloropsis* sp. [39].

**Figure 4:**
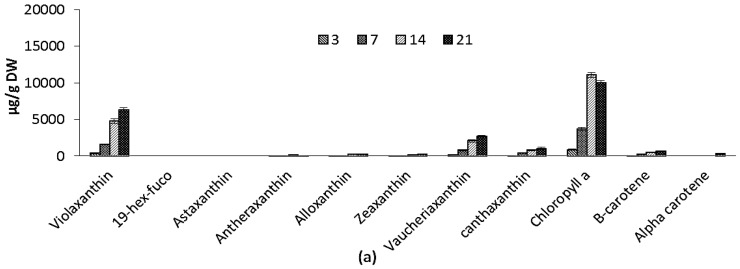

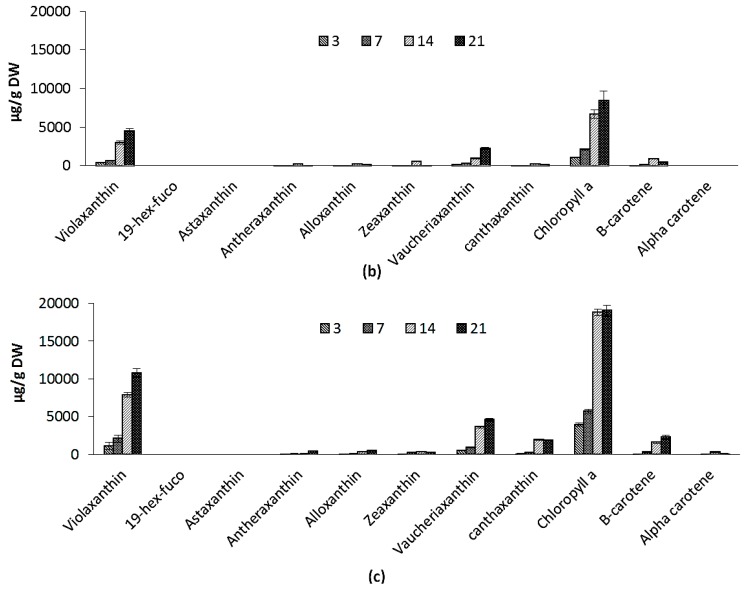
Pigment composition for: (**a**) F/2 experiment; (**b**) ICW + F/2 expeiment; and (**c**) large scale experiment. The standard errors are presented as bars (*n* = 2).

Variations in pigment composition of ICW + F/2 and F/2 experiments are shown in Figure 4a,b. Chlorophyll a (Chl) increased during the course of cultivation from 867 ± 84.0 µg/g to 10,030 ± 239 µg/g and from 1048 ± 22.1 µg/g to 8427 ± 419 µg/g for ICW + F/2 and F/2 cultivated microalgae, respectively. Carotenoids include violaxanthin, vaucheriaxanthin esters, alloxanthin, antheraxanthin, cantaxanthin, zeaxanthin and beta carotene in both experiments. During 21 days of experiment, total carotenoids increased from 749 ± 81 µg/g to 11387 ± 874 µg/g and from 797 ± 55 µg/g to 7761 ± 419 µg/g for F/2 and ICW + F/2 cultivated microalgae, respectively. Both cultivation time and growth media significantly influenced the variations of violaxanthin as main carotenoid, while effect of growth media on variations of vaucheriaxanthin was not found significant. The presence of xanthophyll fatty acid esters (mainly vaucheriaxanthin) is characteristic for carotenoid composition in some microalgae such as *Nannochloropsis* spp. [39], and the free-to-esterified vaucheriaxanthin ratio varies in different *Nannochloropsis* spp. from ca. 1:1 to 1:4 [40].

In this study the ratio of free to esterified vaucheriaxanthin was 1:3.9 for F/2 and 1:3.7 for ICW + F/2 experiments. Like non-esterified vaucheriaxanthin, esterified forms may be attributed to pigment-protein complexes of the thylakoid membranes, but they do not participate in light harvesting in *Nannochloropsis* [39], so vaucheriaxanthin will not follow the variations in light harvesting carotenoids. Car/Chl increased during the cultivation from 0.76 to 0.92 for ICW + F/2 and from 0.86 to 1.13 for F/2 experiments. It was reported previously that considerable rise in Car/Chl along with accumulation of FA takes place similarly and is induced by nitrogen starvation, while there were also reports about accumulation of carotenoids in stressed *Nannochloropsis* cells [39,41]. In this study, nitrogen concentration was higher in ICW + F/2 growth media and resulted in higher chlorophylls and lower carotenoids in the biomass, compared to F/2 growth media. So comparatively higher values of Car/Chl in F/2 cultivated samples can be attributed to the lower levels of nitrogen. It has been clearly demonstrated previously, that in the process of chlorophyll biosynthesis, nitrogen (in form of δ-aminolevulinic acid) is involved as a precursor in the synthesis of pyrol ring in chlorophyll structure [40]. In the presence of a carbon source, concentration of chlorophyll(s) can be directly attributed to the amount of nitrogen (in-organic form) in growth media.

### 2.3. Changes in Growth and Biomass Composition of Large Scale Cultivated Nannochloropsis salina

#### 2.3.1. Growth Rate and Biomass Productivity

The growth curve for large scale cultivation of *Nannochloropsis salina* using nutrient composition as 20% ICW + 80% F/2 at pH 8.3 ± 0.2 is shown in Figure 1c. Growth started rapidly, but did not reach the stationary phase. Variation in optical density and biomass productivity can be attributed to temperature variations (Figure 1d), which resulted in sedimentation and inhomogeneity of the culture. The average solar radiation values from 2006 to 2010 in Denmark previously reported as 16 KWh·m^−2^ for October–November, which compared to June–July (75 KWh·m^−2^) is very low [42]. Daily light varied from 11 h to 9 h during the experiment. Both growth rate and biomass productivity can be affected by low temperature and low daily light hour. For example Sforza et al. [14] reported 30% less growth rate and 28% less biomass concentration for *Nannochloropsis salina* cultivated under light: dark cycle compared to continuous illumination. On the other hand, lower biomass concentration will provide a greater possibility for absorption of light energy in flat panel photo-bioreactor narrow tubes (32 mm). Olofsson et al. [18] reported a relatively constant biomass concentration during two years of cultivation of *Nannochloropsis oculata* in large scale outdoor photo bioreactor, with narrow light path (5 cm). In another study, an increase in the light path length of photo bioreactor from 5 to 10 cm significantly decreased the biomass productivity in *Nannochloropsis gaditana* [21]. Chini Zittelli et al. [17] reported that continuous illumination with artificial light resulted in 37% increase in volumetric productivity of *Nannochloropsis* sp. cultivated in flat panel photo bioreactor. Environmental and operational conditions highly affect the growth and lipid production in *Nannochloropsis* sp. [22] *Nannochloropsis salina* can grow in Denmark’s winter time conditions and tolerate the temperature/light variations, but when high biomass productivity (and not chemical composition) is the main target, artificial light will be required. For efficient cultivation of microalgae in a photo bioreactor, optimizing light availability and light intensity is critically required [14,21,22].

#### 2.3.2. Biomass Chemical Composition

Large scale cultivation of *Nannochloropsis salina* influenced the chemical composition of the biomass during the course of cultivation and before reaching the steady state conditions. Cultivation time significantly influenced variations in lipid and protein contents (*p* < 0.05).

Total lipid contents increased from 10.8% ± 1.2% DW to 21.1% ± 1.2% DW, but the rate of accumulation was lower comparing to the laboratory scale experiment with the same growth media, which can be explained with lower light and temperature. Olofsson et al. [18] also reported that variations in total lipid content of *Nannochloropsis oculata* can be explained with both light and temperature variations. Highest and lowest lipid productivity was reported in autumn and winter, respectively. Camacho-Rodríguez et al. [21] reported that an increase in the path length width of photo bioreactor from 5 to 10 cm significantly decreased both lipid contents in *Nannochloropsis gaditana* from 28% to 24% DW [21]. The geometry of flat-panel bioreactor is a key factor for lipid productivity along with environmental and operational parameters such as pH and dilution (harvest) rate [21,22].

In our study protein contents decreased from 46.0% ± 1.3% DW to 33.4% ± 1.7% DW (Figure 2c_1_). In general variations in protein contents depends to the growth stage, environmental conditions such as light and temperature and composition of growth media. Protein content is also species-related, which can explain higher protein contents in our results compared to another study which reported the variations of protein contents in *Nannochloropsis occulata* [20]. San Pedro et al. [22] reported that protein content of biomass remained in the range of 49.3% and 30.9% DW for *Nannochloropsis gaditana* cultivated in outdoor pilot-scale raceway ponds. It has also been shown that light path lengths in a photo bioreactor can affect the protein contents in *Nannochloropsis gadiata,* as protein contents increased significantly when light path length increased from 5 to 10 cm [21]. In this study, average protein contents were reported as 45% DW with highest values in the microalgae cultivated at the summer. It can be concluded that several factors such as reactor design and specifications, dilution (harvest) rate, growth media and environmental factors such as temperature and light affect the protein accumulation. On the other hand, the protein accumulation in microalgae is species-specific [3]. In our study, rate of variation in protein content was lower compared to the results of laboratory scale cultivation (54.1% and 27.4%, respectively) as shown in Figure 1d. This could be explained by the fact that large scale cultivation was done during the autumn–winter season when environmental factors such as lower light and temperature and sufficient nitrogen in the growth media [20,21,22,33] influence the growth and metabolism pathway.

Uptake and utilization of nitrogen are reported to be temperature sensitive [11], so nitrogen level available in the growth media may have to be optimized during the winter season to obtain optimal growth. It can be considered as benefit when protein is the target compound in the biomass.

Large scale cultivation enhanced the concentration of α-tocopherol as it increased to 431 ± 28 µg/g during 21 days of cultivation compared to 109.08 ± 11 in lab scale (Figure 2C_2_). This difference could be explained by higher availability of light (even in autumn-winter season) in flat panel photo-bioreactor because of short path length compared to the batch laboratory reactors. On the other hand our values were still low, compared to data reported by Durmaz et al. for *Nannochloropsis oculata*, which was reported to contain 2325.8 ± 39 µg/g α-tocopherol [35]. This lower concentration can be explained by seasonal low photoperiod, which was demonstrated as an important factor, so it could be suggested that in spring-summer season α-tocopherol content will be increased with the same growth media. Durmaz et al. [35] also reported that concentration of tocopherols increased significantly in stationary phase as a response to the nitrogen starvation.

Variations in fatty acid composition resembled the laboratory scale findings for C16:0 and total saturates, which decreased during the cultivation time from 24.9% ± 2.28% TFA to 17.4% ± 0.24% TFA and from 32.0% ± 3.10% TFA to 21.2% ± 0.74% TFA, respectively (Table 4). EPA and total *n*-3 fatty acids increased accordingly, from 23.9% ± 1.78% TFA to 44.2% ± 2.30% TFA and from 27.8% ± 2.30% TFA to 48.1% ± 2.43% TFA, respectively. Opposite of laboratory scale results, variation in C16:1 was not significant. Results of EPA reported in our study are among the highest ever reported for *N. salina* in both laboratory and large scale [11,12,13,15,18,19,20,25,32,33]. Several studies reported that low temperature and short day light, which is equal to less solar energy, could enhance the EPA accumulation in *Nannochloropsis* sp. [15,18,19,20,21,22]. The geometry of a photo-bioreactor can also affect the EPA productivity in *Nannochloropsis* sp. [21]. A significant decrease in EPA productivity was obtained when the light path length in the photo-bioreactor increased from 5 to 10 cm, while the highest EPA productivity was obtained in winter [21]. Low temperatures can decrease the cell membrane fluidity, and microalgae counteracted this environmental effect by increased synthesis of polyunsaturated fatty acids such as EPA [11]. It can be concluded that low temperature (12.0 ± 1.5 °C) during large scale cultivation enhanced the accumulation of EPA in *Nannochloropsis salina*, compared to laboratory scale results for the experiment with the same growth media (ICW + F/2). Laboratory scale experiments were carried out at 20 ± 2 °C.

Variations in amino acid composition of large scale cultivated *N. salina* are shown in Figure 3c. To our knowledge not the same study concerning the variations in amino acid composition in the *Nannochloropsis* sp. cultivated in large scale. Glutamate was rated as the main amino acid, while concentration of lysine, arginine, and leucine were nearly the same. It could be concluded that the variations in amino acids in large scale followed the variation in protein contents in laboratory scale experiments. The presented amino acid composition for large scale cultivated *Nannochloropsis salina* was an ideal composition for aquaculture feed formulas [38]. Total amino acid content also decreased during the cultivation with a gentler slope than ICW + F/2 laboratory scale experiment (Figure 3d).

Pigment composition of large scale cultivated *Nannochloropsis salina* resembled the results, which were discussed previously for laboratory scale cultivated species. Variations in pigment composition are shown in Figure 4c. Chlorophyll content increased during the course of cultivation from 3944 ± 200 µg/g to 19,020 ± 720 µg/g. During 21 days of experiment, total carotenoids increased from 2038 ± 48 µg/g to 20,797 ± 1048 µg/g. Amounts of beta-carotene increased significantly from 26.9 ± 48 µg/g to 2284 ± 234 µg/g. Other carotenoids including vaucheriaxanthin, canthaxanthin, and zeaxanthin also increased during the cultivation time. Free to esterified vaucheriaxanthin ratio was 1:4.2. Car/Chl increased during the cultivation from 0.5 to 1.1. This finding can be explained by environmental stresses such as low temperature and photoperiod, which may influence the light harvesting pigments, i.e., mainly violaxanthin in *Nannochloropsis* sp. [15,36].

## 3. Materials and Methods

### 3.1. Chemicals and Reagents

Standards of fatty acids, amino acids and tocopherols were purchased from Sigma (St. Louis, MO, USA) and Fluka (Deisenhofen, Germany). Standards of pigments were purchased from DHI (Hørsholm, Denmark). HPLC grade acetonitrile, heptane, isopropanol, methanol and acetone were purchased from Sigma and Fluka. HPLC grade water was prepared at DTU Food using Milli-Q^®^ Advantage A10 water deionizing system from Millipore Corporation (Billerica, MA, USA).

### 3.2. Growth Media

Industrial process water (ICW) was collected from effluent stream of an anaerobic methanogenic conversion reactor (Novozymes plant, Kalundborg, Denmark). Batches of industrial process water was filtered using an out-side-in dynamic cross flow microfiltration BioBooster system from Grundfos A/S (Bjerringbro, Denmark) equipped with 0.2 µm ceramic disc filters and stored at −20 °C prior to use. Chemical composition of the industrial process water is shown in Table 1. The basic culture medium was a conventional algae F/2 growth media powder based on Guillard & Ryther [24], which was purchased from Varicon Aqua solutions (Worcs, UK). Salt brine was prepared from refined salt (Scan salt A/S, Kolding, Denmark) and UV-sterilized and micro-filtrated tap water, which was added to the growth media in order to achieve a final salinity of 25 g/L.

### 3.3. Laboratory Scale Cultivation of Microalgae

Microalgae strain *Nannochloropsis salina* (Strain Number: 40.85) was obtained from culture collection of algae (SAG), University of Gottingen. The strain was cultivated in 1–5 L Schott bottles, while shaking gently in an orbital platform shaker (Heidolf instruments GmbH, Schwabach, Germany). During the cultivation, all reactors were continuously aerated with 2% carbon dioxide/air mixture under fluorescent lights with an intensity of 100 µmol photon m^−2^·s^−1^ and 12:12 (day:night) photoperiod. Light intensity was measured using a Li-190 quantum sensor (LI-COR, Inc., Lincoln, NE, USA). Online monitoring and control of pH was performed by Milwaukee MC-122-pH controller (Milwaukee Electronics, Szeged, Hungary) equipped with solenoid valve to control the stream of CO_2_. Control of temperature was done by an aluminum plate connected to cold water circulation system and the temperature was kept constant at 23 ± 2 °C. Preliminary experiments were performed to explore growth rates at different levels of substitution of F/2 stand growth media with ICW (20%, 30%, and 40%), and pH (6.3 ± 0.2, 7.3 ± 0.2, and 8.3 ± 0.2). Table 2 shows the type and amounts of nitrogen in each growth media. Cultivation was repeated for *N. salina* on 20% ICW + 80% F/2 and standard F/2 media in 10 L Schott bottles at pH 8.3 ± 0.2 and with the same conditions as mentioned before. During the cultivation and at each sampling point, 0.25 L of culture was taken and samples were centrifuged at 10,000 g. Then resulting biomass pellet was washed twice with deionized water. Resulting biomasses were freeze dried immediately to moisture content less than 1% DW. Measurement of moisture was done by an AD 4714A moisture analyzer (A&D Company, Tokyo, Japan). Samples stored at −20 °C prior to chemical analysis.

*N. salina* cultivated on 20% ICW + 80% F/2 was transferred to Kalundborg microalgae facility (Kalundborg, Denmark) for large scale experiment.

### 3.4. Large-Scale Cultivation of Microalgae

**Figure 5:**
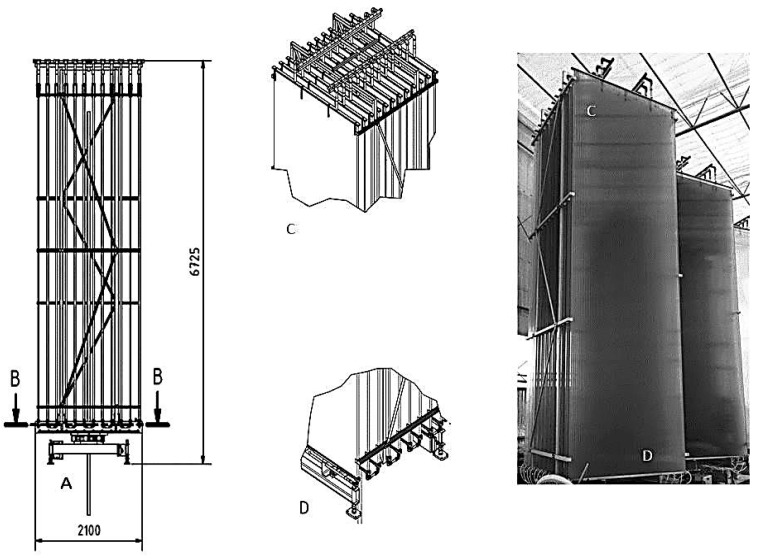
Schematic of flat panel photo-bioreactor which was used for the large scale cultivation experiment. (**A**) Rotating system; (**B**) ground level; (**C**) top part; (**D**) bottom part. Every unit includes 12 sheets. Sheet dimensions: width 2100 mm; height 5600 mm and depth 32 mm.

Large scale cultivation was done at the Kalundborg microalgae facility using flat panel photo-bioreactor system, type hanging garden from Ecoduna produktions GmbH (Bruck/Leitha, Austria). The photo-bioreactors were designed to track the sun thereby allowing the algae to grow most efficiently on natural sunlight. No artificial light was used during the large scale experiment. The system base unit includes 12 hollow chamber sheets (width 2100 mm; height 5600 mm and depth 32 mm) which are assembled on guide rails. The microalgae suspension was transferred into the sheets and was continuously circulated through the module. Each sheet was sealed off with a plastic cover on the top and with a deflecting profile on the bottom and supplied with nutrient stream from the bottom deflecting profile on the lateral input. The nutrient stream was pumped through the sheets by means of hydrostatic pressure and the gas lift effect, as a result, no additional energy is required. In these specific reactors, surface hit by sunlight is multiplied and the light is being distributed so the irradiation will never be too high. All of the sheets of a module were interconnected via a hose system thus ensuring the obstruction-free transfer of the nutrient solution. Details of the flat panel bioreactor are shown in Figure 5. Air stream was filtered through 0.2 µm membrane filter and mixed with pure CO_2_ from Cylinder (purity 99.5% *v*/*v*). A mixture of air and 2% *v*/*v* CO_2_ was then introduced from below to ensure gentle but effective mixing of nutrients and algae to control the pH which was adjusted on 8.5 ± 0.5. This Gas stream drives a gentle liquid flow (1 L·min^−1^ via an “air-lift effect” so the system does not require pump unit. Cultivation was done in batch mode and two separate units were used. At each sampling point, 2 L of culture was taken and samples were centrifuged at 10,000 g. Biomass samples were washed twice with distilled water and then immediately freeze dried and stored at −20 °C prior to the analysis. Cultivation was done during the period of October–November 2015. The culture was kept at steady state basis, by continuous biweekly harvest and feeding of new growth media after day 14.

### 3.5. Analytical Methods

#### 3.5.1. Growth Rate

Growth rate was monitored by daily detection of optical density at 750 nm.

#### 3.5.2. Protein and Amino Acids

The protein content in the microalgae samples was estimated using a modified Micro biuret method [43] with some modification. One mL of 0.5 M NaOH aqueous solution was added to 10 mg of sample and extraction was carried out at 80 °C for 10 min. Samples were centrifuged at 5000 g and supernatants were moved to new tubes. Extraction was repeated 3 times and all extracts were mixed together before analysis. For protein content estimation, 200 µL of copper sulphate (0.21% CuSO_4_·5H_2_O in 30% NaOH aqueous solution) was mixed with 300 µL of the extract and 500 µL of water. Color formation was monitored at 310 nm. Bovine serum albumin (BSA) was used as the standard for calibration curves.

The amino acid composition was analyzed using EZ:faast™ amino acid analysis kit (Phenomenex Inc., Torrance, CA, USA). For protein hydrolysis around 30 mg of microalgae was weighted in microwave glass vials. The samples were first overlaid with nitrogen and then hydrolyzed in 6 M HCl in microwave oven (Anton Paar GmbH, Graz, Austria) for 60 min at 110 °C. Following individual cleanup-step for removing matrix interference, amino acids derivatization was done. The amino acid composition was determined by liquid chromatography with a Agilent 1100 series LC/MSD Trap mass spectrometry (Agilent technologies, Hørsholm, Denmark) using EZ:faast™ LC-MS column (250 × 3.0 mm, Phenomenex, Torrance, CA, USA).

#### 3.5.3. Lipids, Fatty Acid and Tocopherols

Lipids were extracted in chloroform, methanol and water, as described by Bligh and Dyer using 200 mg of dried microalgae biomass [44].

Fatty acid profile was analyzed according to the method (FAME) based on the AOCS official method [45]. Around 1 g of Bligh and Dyer extract was weighted in methylation glass tube and was evaporated to dryness under a gentle stream of nitrogen. 100 μL of internal standard solution (2% *w*/*v* C23:0 in heptane), 200 μL of heptane with BHT (0.01% *w*/*v*), 100 μL of toluene and 1 mL of borontriflouride in methanol (BF_3_-MeOH) was added. Samples were mixed and methylated in microwave oven (Microwave 3000 SOLV, Anton Paar, Ashland, VA, USA) for 10 min at 100 °C and power of 500 watts and then cooled down for 5 min. 1 mL of saturated salt water (NaCl) and 0.7 mL of heptane with BHT were added. After the separation of heptane the upper phase of the sample (around 0.7 mL) was transferred into vials. Samples were analyzed by gas chromatography system (HP-5890 A, Agilent Technologies, Santa Clara, CA, USA). Fatty acid methyl esters were separated and detected by the GC column Agilent DB wax 127-7012 (10 μm × 100 μm × 0.1 μm), from Agilent technologies (Santa Clara, CA, USA).

Analysis of tocopherols and toco-trienols was done by using LC-FLD. 3 g of Bligh and Dyer extract were weighted and evaporated to dryness under a gentle stream of nitrogen. Dry sample was mixed with 1 mL of heptane and then transferred to HPLC vials. Analysis was done based on the AOCS official method [45] using Agilent 1100 Liquid Chromatograph (Agilent Technologies, Santa Clara, CA, USA), equipped with a fluorescence detector, with the excitation wavelength set at 290 nm and emission wavelength at 330. The separation was carried out by a Spherisorb column 150 mm × 46 mm × 3 µm particle size (Waters Corporation, Milford, MA, USA), using mixture of isopropanol and heptane (0.5:99.5) as mobile phase.

#### 3.5.4. Pigments

Extraction and analysis of the pigments was done using the method described by Safafar et al. [34]. Samples were extracted by methanol containing BHT (butylated hydroxyl toluene) in sonication bath (Branson ultrasonics, Danbury, CA, USA) at a temperature lower than 5 °C for 15 min. Samples were analyzed by HPLC using Agilent 1100 Liquid Chromatograph equipped with a DAD. The separation was carried out in a Zorbax Eclipse C8 column 150 mm × 46 mm × 3.5 μm from Phenomenex. The mobile phase was a mixture of solvent A (70% methanol + 30% of 0.028 M tertiary butyl ammonium acetate in water) and solvent B (methanol) at a flow rate of 1.1 mL·min^−1^. Total acquisition time was 40 min. Identification of peaks and calibration was done by individual standard for each pigment. Detection for carotenoids, chlorophylls and internal standard (BHT) was done at 440 nm, 660 nm, and 280 nm, respectively.

#### 3.5.5. Statistical Analysis

All experiments were repeated two times independently, and data were recorded as the mean. Results were evaluated using ANOVA to test the effect of time and growth media for lab scale and time for large scale experiments. Multiple comparison procedure based on Fisher’s least significant difference (LSD) was used to discriminate among the means at the 95.0% confidence level. All statistical analyses were done by STATGRAPHICS software, version Centurion XVI (Stat point Technologies Inc., Warrenton, VA, USA).

## 4. Conclusions

Cultivation of *N. salina* in a mixture of industrial process water and F/2 standard growth media enhanced the EPA content compared to F/2 growth media. Large-scale cultivation of *Nannochloropsis salina* in a flat panel photo-bioreactor confirmed the laboratory-scale findings. The algae growth rate at winter condition of Denmark was slow, but results revealed that large-scale cultivation of *Nannochloropsis salina* in these conditions could improve the nutritional properties such as EPA, protein, α-tocopherol and carotenoids compared to culturing in lab scale. *Nannochloropsis salina* is a good candidate for large-scale cultivation in the autumn–winter climate in Denmark. Resulting biomass from *Nannochloropsis salina* could be a good source of EPA, amino acid, tocopherols and carotenoids for the aquaculture feed industry. The biomass could also be a rich source of these bioactive compounds for the food industry, but this may require further extraction and fractionation of the biomass.

## Figures and Tables

**Table 1:** Chemical composition of industrial wastewater.

Item	Unit	Amount
pH	-	8.1
Suspended solids	mg/L	20
Total N	mg/L	190
Ammonia + ammonium-N	mg/L	150
Nitrite + nitrate	mg/L	<0.1
Total P	mg/L	11
Sulphate	mg/L	3.6
Total Alkalinity	mmol/L	62.5
EDTA	mg/L	<0.5
Sodium(Na)	mg/L	1500
Copper (Cu)	µg/L	3.4
Iron (Fe)	mg/l	0.23

**Table 2:** Type and amounts of nitrogen in each growth media.

Growth Media *	${\text{NO}}_{3}^{-}$	${\text{NH}}_{4}^{+}$	Total N	Total P
F/2	75	-	75	5.0
20% ICW + 80% F/2	60	30	90	6.2
30% ICW + 70% F/2	52	45	97	6.8
40% ICW + 60% F/2	45	60	105	7.7

* All values are in mg/L.

**Table 3:** Fatty acid composition of *N. salina* grown on F/2 and ICW + F/2 growth media in laboratory scale. Different letters indicate significant differences (*p* < 0.05).

Cultivation Time (Day)	3		7		14		21	
Fatty Acid	F/2	ICW + F/2	F/2	ICW + F/2	F/2	ICW + F/2	F/2	ICW + F/2
14:0	1.45 ± 0.44	3.22 ± 0.50	3.52 ± 0.12	0.51 ± 0.05	1.40 ± 0.13	0.56 ± 0.30	2.05 ± 0.50	3.53 ± 0.13
15:0	0.04 ± 0.00	nd	nd	nd	0.54 ± 0.01	1.10 ± 0.40	0.30 ± 0.09	1.46 ± 0.03
16:0	48.8 ± 0.59 ^a^	43.3 ± 0.98 ^b^	32.9 ± 0.53 ^c^	31.9 ± 0.77 ^c^	23.7 ± 0.40 ^d^	20.6 ± 0.64 ^de^	21.3 ± 0.30 ^de^	19.1 ± 0.40 ^e^
16:1 (*n*-7)	30.3 ± 0.40 ^a^	30.9 ± 0.10 ^a^	24.0 ± 0.09 ^b^	20.4 ± 0.02 ^c^	25.1 ± 0.51 ^b^	23.0 ± 0.02 ^b^	31.9 ± 0.66 ^a^	17.7 ± 0.51 ^c^
16:2 (*n*-4)	0.18 ± 0.01	0.23 ± 0.02	1.24 ± 0.00	0.65 ± 0.00	0.33 ± 0.01	0.76 ± 0.07	0.64 ± 0.02	0.38 ± 0.01
16:3 (*n*-4)	0.11 ± 0.03	3.57 ± 0.09	0.61 ± 0.02	0.56 ± 0.03	0.75 ± 0.04	0.63 ± 0.08	0.24 ± 0.05	1.01 ± 0.04
16:4 (*n*-1)	0.36 ± 0.22	3.35 ± 0.08	0.45 ± 0.04	0.14 ± 0.19	0.44 ± 0.01	0.28 ± 0.10	0.43 ± 0.03	0.66 ± 0.06
18:0	2.35 ± 0.20	1.69 ± 0.19	5.32 ± 0.09	1.82 ± 0.06	2.20 ± 0.01	2.10 ± 0.06	0.51 ± 0.10	1.86 ± 0.07
18:1 (*n*-9)	7.03 ± 0.23	3.31 ± 0.06	9.43 ± 0.08	5.61 ± 0.06	9.06 ± 0.20	6.53 ± 0.06	4.22 ± 0.06	4.38 ± 0.20
18:1 (*n*-7)	0.75 ± 0.20	0.00 ± 0.06	0.45 ± 0.08	6.31 ± 0.60	7.52 ± 0.21	7.50 ± 0.45	0.57 ± 0.06	3.32 ± 0.21
18:2 (*n*-6)	1.13 ± 0.01	0.74 ± 0.06	1.45 ± 0.03	1.35 ± 0.06	2.23 ± 0.08	1.67 ± 0.07	1.63 ± 0.03	1.46 ± 0.09
18:3(*n*-3)	0.64 ± 0.10	0.50 ± 0.01	0.49 ± 0.04	0.46 ± 0.03	0.04 ± 0.02	nd	0.30 ± 0.01	nd
20:1(*n*-7)	0.10 ± 0.00	nd	1.20 ± 0.12	nd	0.10 ± 0.01	nd	0.10 ± 0.01	nd
20:4 (*n*-6)	1.21 ± 0.20	0.73 ± 0.06	1.92 ± 0.05	4.82 ± 0.08	3.82 ± 0.07	5.63 ± 0.08	2.33 ± 0.06	4.91 ± 0.07
20:3 (*n*-3)	0.22 ± 0.08	0.33 ± 0.08	1.57 ± 0.04	0.26 ± 0.02	nd	0.31 ± 0.02	0.17 ± 0.08	0.93 ± 0.02
20:4 (*n*-3)	0.10 ± 0.00	0.33 ± 0.02	0.43 ± 0.03	nd	0.31 ± 0.08	nd	0.10 ± 0.00	nd
20:5 (*n*-3)	4.95 ± 0.78 ^a^	7.47 ± 0.23 ^ab^	10.9 ± 0.11 ^b^	22.2 ± 0.21 ^c^	19.9 ± 0.42 ^c^	26.2 ± 0.25 ^d^	32.0 ± 0.82 ^e^	37.1 ± 0.77 ^f^
22:5 (*n*-3)	0.66 ± 0.05	nd	0.40 ± 0.01	0.26 ± 0.04	1.35 ± 0.05	0.14 ± 0.03	0.66 ± 0.03	0.27 ± 0.05
22:6 (*n*-3)	0.46 ± 0.09	0.15 ± 0.04	0.24 ± 0.02	1.04 ± 0.40	nd	0.69 ± 0.40	0.19 ± 0.02	0.16 ± 0.05
∑ SAFA	51.6 ± 3.25 ^a^	48.3 ± 2.33 ^a^	41.8 ± 0.63 ^b^	34.2 ± 1.25 ^b^	27.9 ± 0.51 ^c^	24.4 ± 1.44 ^c^	24.2 ± 1.63 ^c^	25.9 ± 0.62 ^c^
∑ *n*-3	6.92 ± 2.41 ^a^	8.77 ± 0.45 ^a^	13.6 ± 0.23 ^b^	24.3 ± 0.70 ^c^	21.66 ± 0.66 ^c^	27.4 ± 0.74 ^d^	33.37 ± 1.0 ^e^	38.6 ± 1.00 ^f^

Data are shown as % of fatty acid in total fatty acid composition. Total saturated fatty acids (Σ SAFA), *n*-3 poly unsaturated fatty acid (∑ *n*-3); nd, not detected. Different letters in the same row represent significant difference (*p* < 0.05) for 16:0, 16:1 (*n*-7), 20:5 (*n*-3), total SAFA and *n*-3 fatty acids.

**Table 4:** Fatty acid composition of *N. salina* cultivated in flat-panel photo-bioreactor. Different letters indicate significant differences (*p* < 0.05).

Cultivation Time (Day)	3	7	14	21
Fatty Acid				
14:0	5.28 ± 0.57	4.20 ± 0.50	3.58 ± 0.37	3.37 ± 0.37
15:0	0.67 ± 0.44	0.38 ± 0.04	0.26 ± 0.04	0.24 ± 0.04
16:0	24.9 ± 2.28 ^a^	19.9 ± 0.59 ^b^	18.5 ± 0.19 ^b^	17.4 ± 0.24 ^b^
16:1 (*n*-7)	26.5 ± 2.59 ^a^	28.7 ± 0.30 ^a^	28.1 ± 0.44 ^a^	26.4 ± 0.19 ^a^
16:2 (*n*-4)	0.18 ± 0.03	0.23 ± 0.02	0.10 ± 0.02	0.10 ± 0.00
16:3 (*n*-4)	0.20 ± 0.01	0.45 ± 0.02	0.22 ± 0.03	0.10 ± 0.02
16:4 (*n*-1)	0.44 ± 0.03	1.01 ± 0.02	0.50 ± 0.02	0.21 ± 0.02
18:0	1.91 ± 0.22	0.37 ± 0.08	0.26 ± 0.03	0.47 ± 0.02
18:1 (*n*-9)	6.06 ± 0.76	3.93 ± 0.19	1.87 ± 0.10	1.76 ± 0.21
18:1 (*n*-11)	2.62 ± 0.20	1.31 ± 0.06	0.94 ± 0.03	0.24 ± 0.00
18:2 (*n*-6)	2.39 ± 0.23	1.30 ± 0.06	0.16 ± 0.01	0.88 ± 0.03
18:3 (*n*-3)	0.10 ± 0.01	nd	nd	nd
20:1 (*n*-9)	0.10 ± 0.02	0.11 ± 0.01	nd	nd
20:1(*n*-7)	0.24 ± 0.02	nd	0.14 ± 0.01	nd
20:4 (*n*-6)	0.05 ± 0.02	nd	nd	0.13 ± 0.01
20:3 (*n*-3)	1.10 ± 0.03	6.45 ± 0.06	3.38 ± 0.05	2.75 ± 0.03
20:4 (*n*-3)	0.61 ± 0.01	0.10 ± 0.08	1.02 ± 0.34	0.91 ± 0.01
20:5 (*n*-3)	23.9 ± 1.78 ^a^	30.1 ± 1.21 ^b^	39.3 ± 1.10 ^c^	44.2 ± 2.30 ^d^
22:1 (*n*-11)	nd	0.16 ± 0.03	0.10 ± 0.03	0.12 ± 0.03
21:5 (*n*-3)	1.82 ± 0.39	0.81 ± 0.35	1.03 ± 0.01	0.09 ± 0.01
22:5 (*n*-3)	0.32 ± 0.05	0.23 ± 0.03	0.07 ± 0.01	0.06 ± 0.02
22:6 (*n*-3)	nd	0.12 ± 0.20	0.10 ± 0.04	0.10 ± 0.01
∑ SAFA	32.0 ± 3.10 ^a^	24.5 ± 1.21 ^b^	22.3 ± 0.72 ^b^	21.2 ± 0.74 ^b^
∑ *n*-3	27.8 ± 2.30 ^a^	37.7 ± 2.00 ^b^	44.9 ± 1.52 ^bc^	48.1 ± 2.43 ^c^

Data are shown as % of fatty acid in total fatty acid composition. Total saturated fatty acids (Σ SAFA), *n*-3 poly unsaturated fatty acid (∑ *n*-3); nd, not detected. Different letters in the same row represent significant difference (*p* < 0.05) for 16:0, 16:1 (*n*-7), 20:5 (*n*-3), total SAFA and *n*-3 fatty acids.

## Abbreviations

The following abbreviations are used in this manuscript:

EPA	Eicosa pentanoic acid
ICW	Industrial process water
BHT	Butylated hydroxyl toluene
FPPBR	Flat panel photo bioreactor
